# Supplementary figures and images for: Reproductive performance of lumpfish (Cyclopterus lumpus, L. 1758) females: Effects of integrated photoperiod and temperature manipulations on sexual maturation and spawning
Source: PLoS One. 2024 Oct 15;19(10):e0311735. doi: 10.1371/journal.pone.0311735 (PMC11478831; doi:10.1371/journal.pone.0311735)

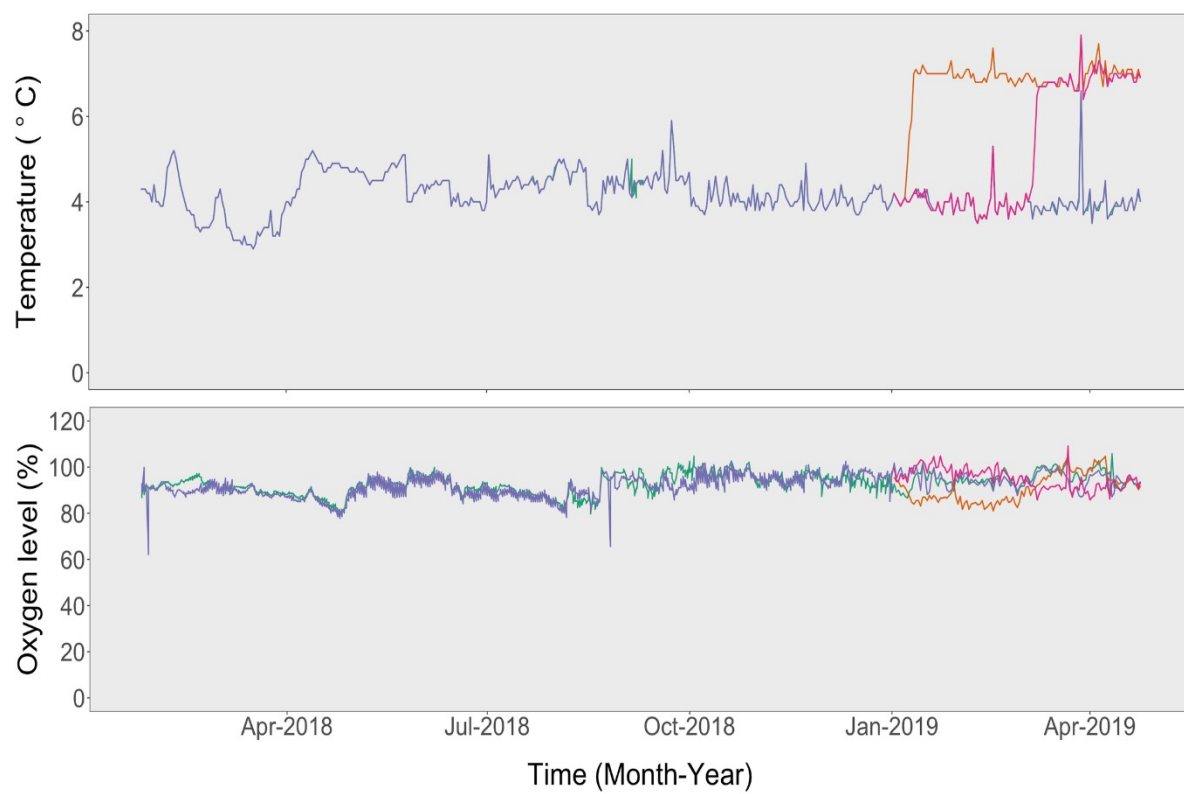

Supplement: S1 Fig — (PDF) [file pone.0311735.s001.pdf]

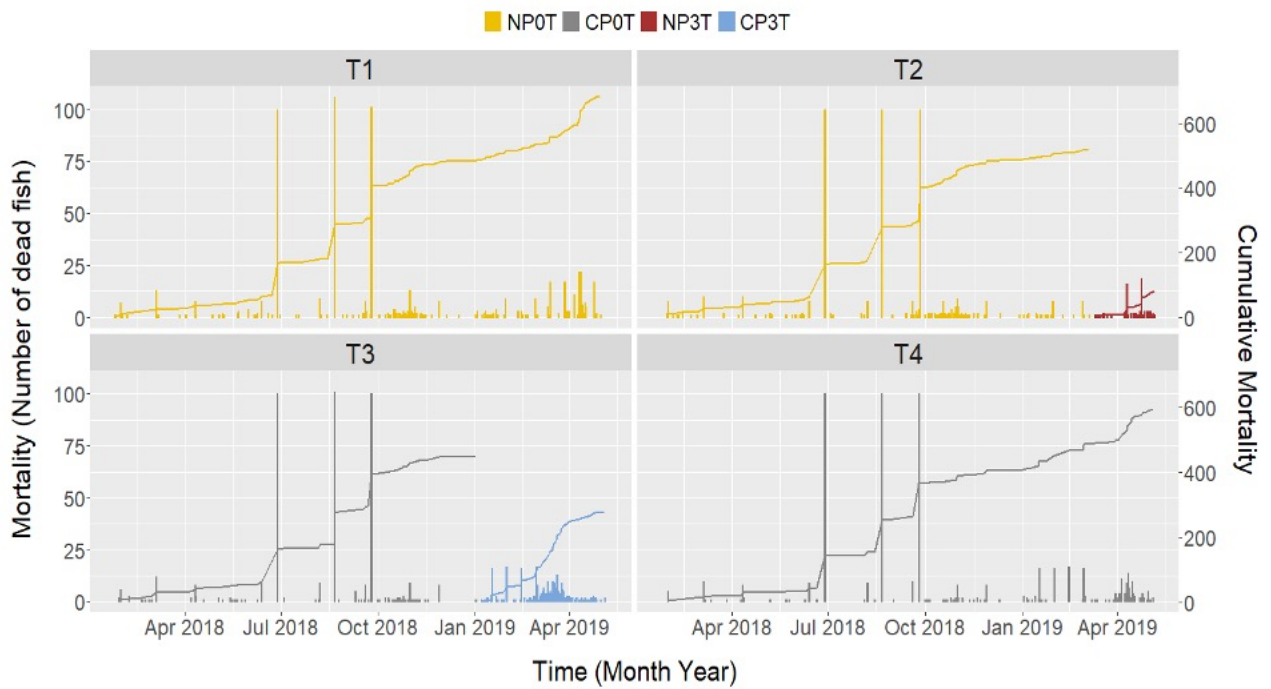

Supplement: S2 Fig — Condition factors of lumpfish females from different photoperiods (until 03-Jan-19) and different photoperiod and temperature combinations (from 03-Jan-19). NP0T: natural photoperiod at ambient temperature, CP0T: compressed photoperiod at ambient temperature, NP3T: natural photoperiod at elevated temperature, CP3T: compressed photoperiod at ambient temperature. The red rectangle on 03-Jan-2019 indicates the start of temperature elevation, and the red vertical line separates the periods before and after temperature elevation. Values are mean ± S.E.M and statistical significances between groups at a sampling point are indicated by “*” or “ns”. Statistical differences between sampling points are graphically displayed in supporting information on S8–14 Figs. For trend visualization, refer to (S6 Fig), which presents the data as a line graph. (PDF) [file pone.0311735.s002.pdf]

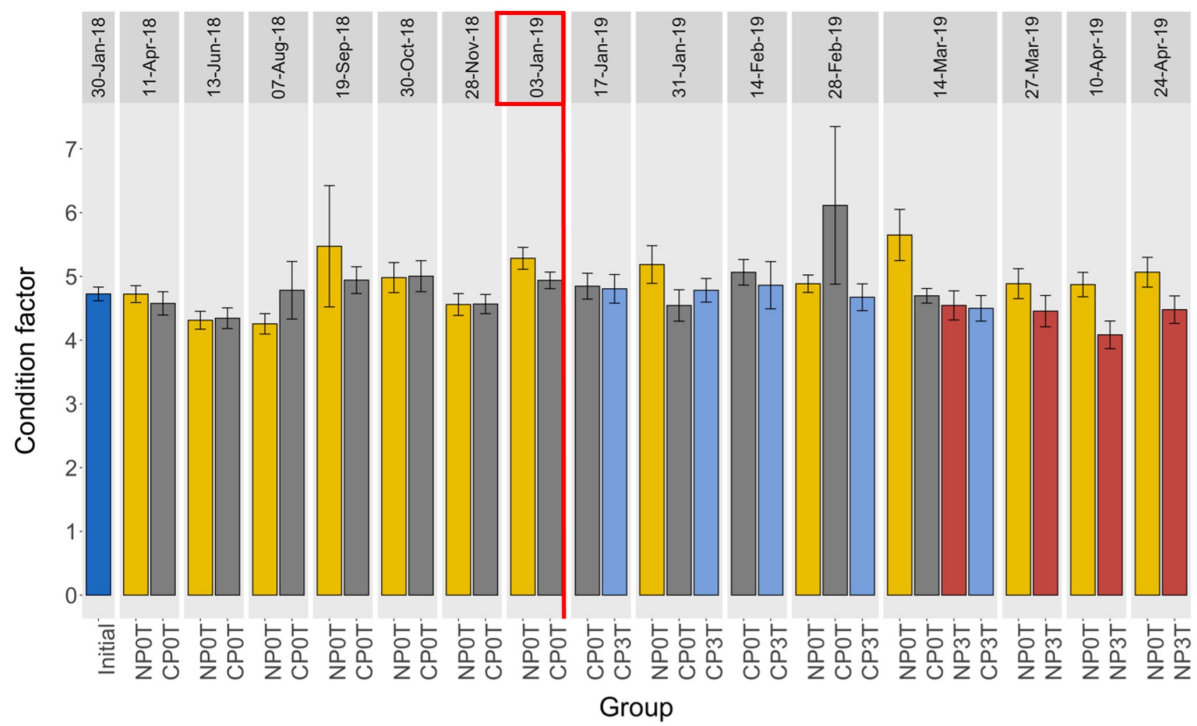

Supplement: S3 Fig — Number of dead fish (left y aixs) and cumulative number of dead fish (right y axis) in different photothermal regimes. T1, T2, T3 and T4 represent Tank 1, Tank 2, Tank 3, and Tank 4, respectively. (PDF) [file pone.0311735.s003.pdf]

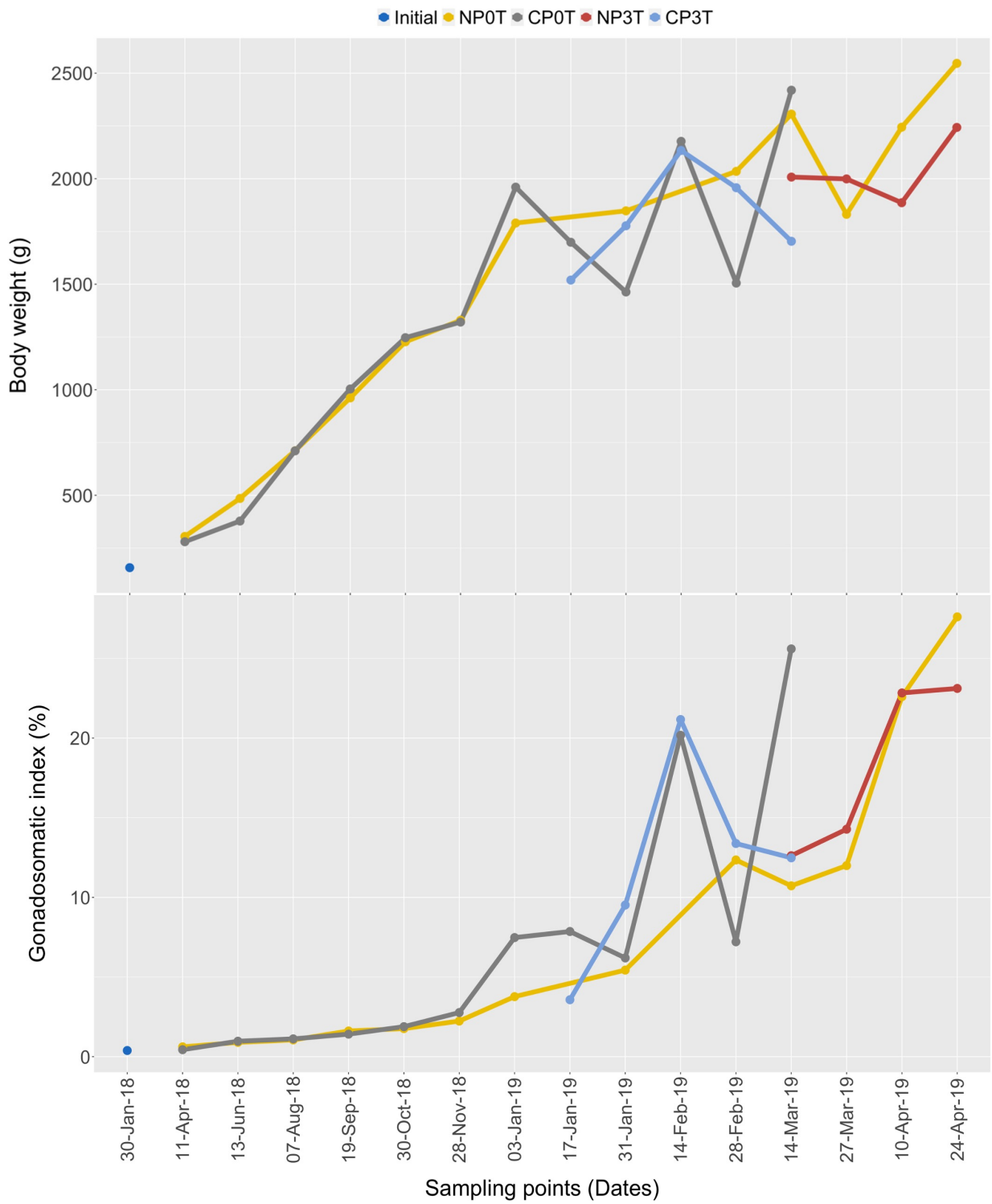

Supplement: S4 Fig — Body weights and gonadosomatic indices of lumpfish females from different photoperiods (until 03-Jan-19), and different photoperiod and temperature combinations (from 03-Jan-19). NP0T: natural photoperiod at ambient temperature, CP0T: compressed photoperiod at ambient temperature, NP3T: natural photoperiod at elevated temperature, CP3T: compressed photoperiod at ambient temperature. (PDF) [file pone.0311735.s004.pdf]

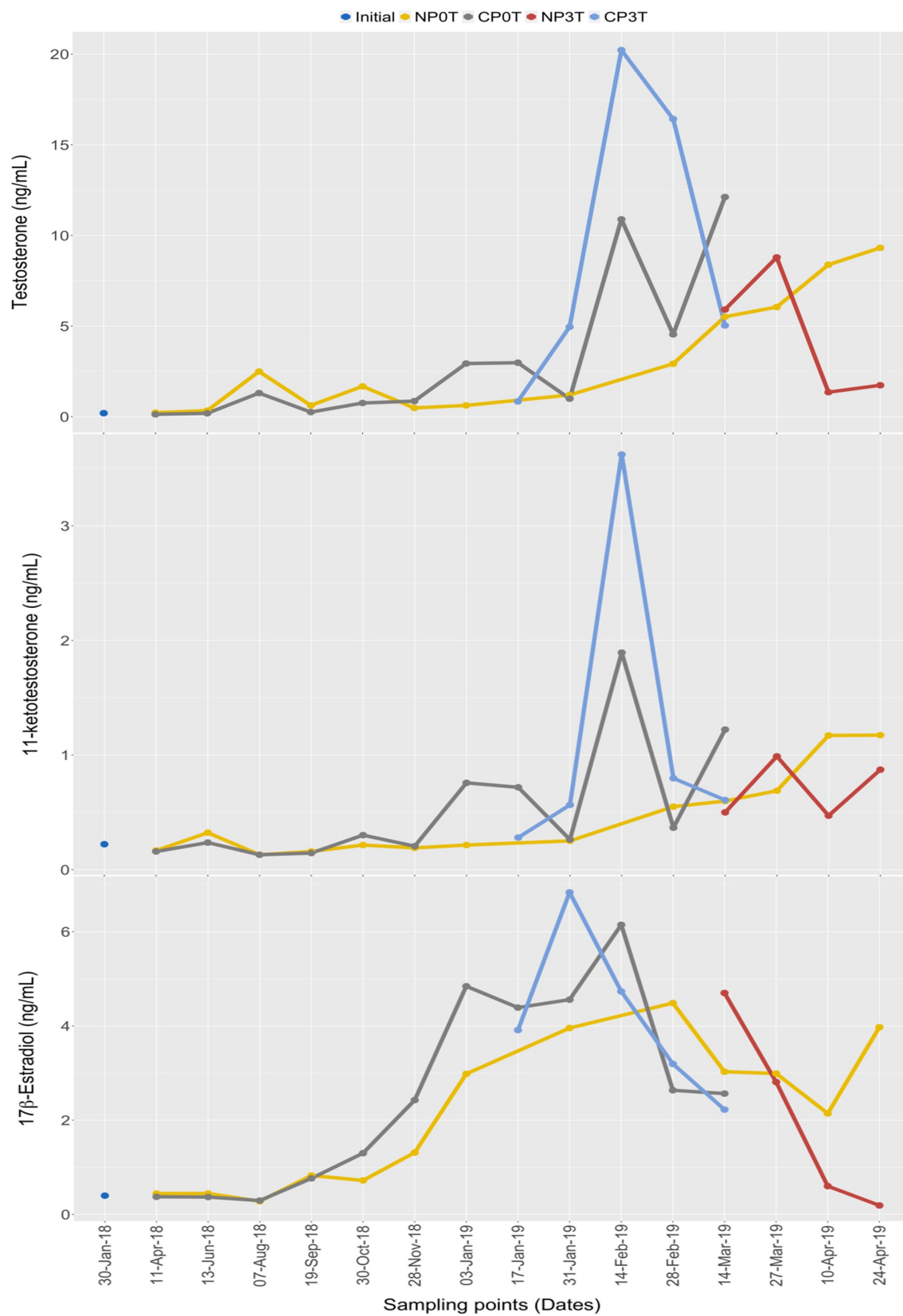

Supplement: S5 Fig — Blood plasma levels of testosterone (top), 11-ketotestosterone (middle) and 17β-estradiol (bottom) in lumpfish females from different photoperiods (until 03-Jan-19), and different photoperiod and temperature combinations (from 03-Jan-19). NP0T: natural photoperiod at ambient temperature, CP0T: compressed photoperiod at ambient temperature, NP3T: natural photoperiod at elevated temperature, CP3T: compressed photoperiod at ambient temperature. (PDF) [file pone.0311735.s005.pdf]

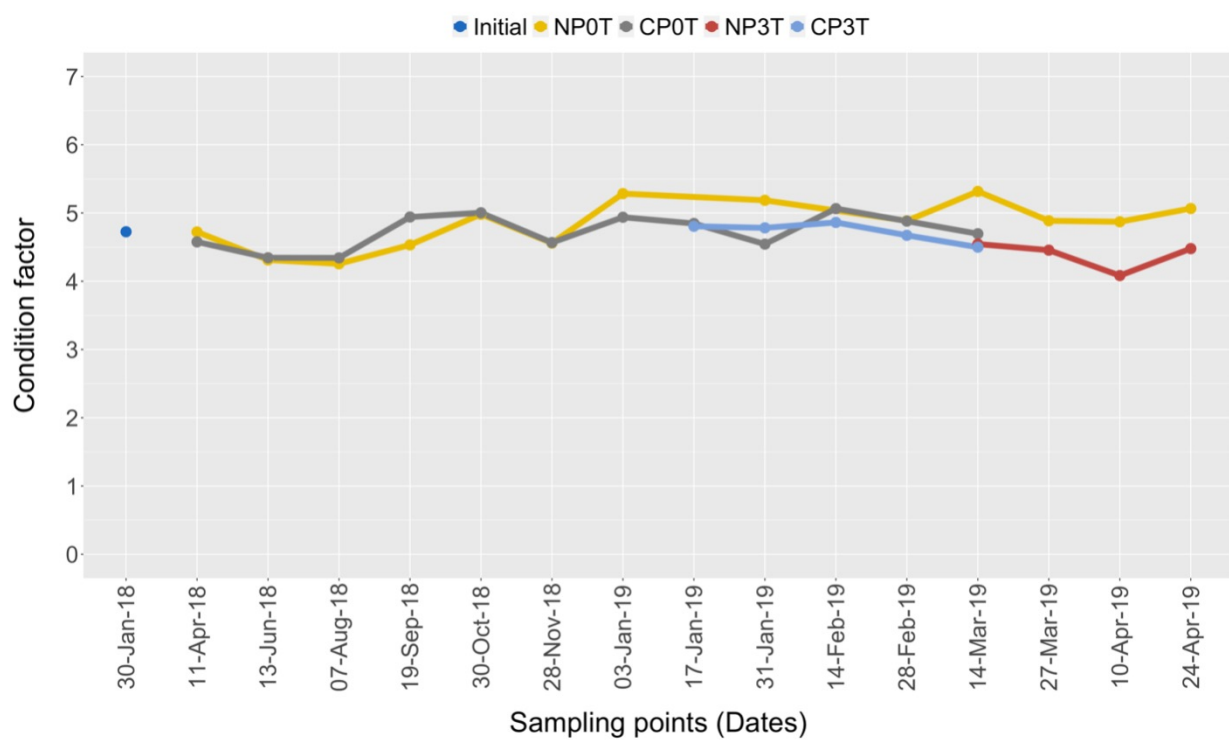

Supplement: S6 Fig — Condition factors of lumpfish females from different photoperiods (until 03-Jan-19) and different photoperiod and temperature combinations (from 03-Jan-19). NP0T: natural photoperiod at ambient temperature, CP0T: compressed photoperiod at ambient temperature, NP3T: natural photoperiod at elevated temperature, CP3T: compressed photoperiod at ambient temperature. (PDF) [file pone.0311735.s006.pdf]

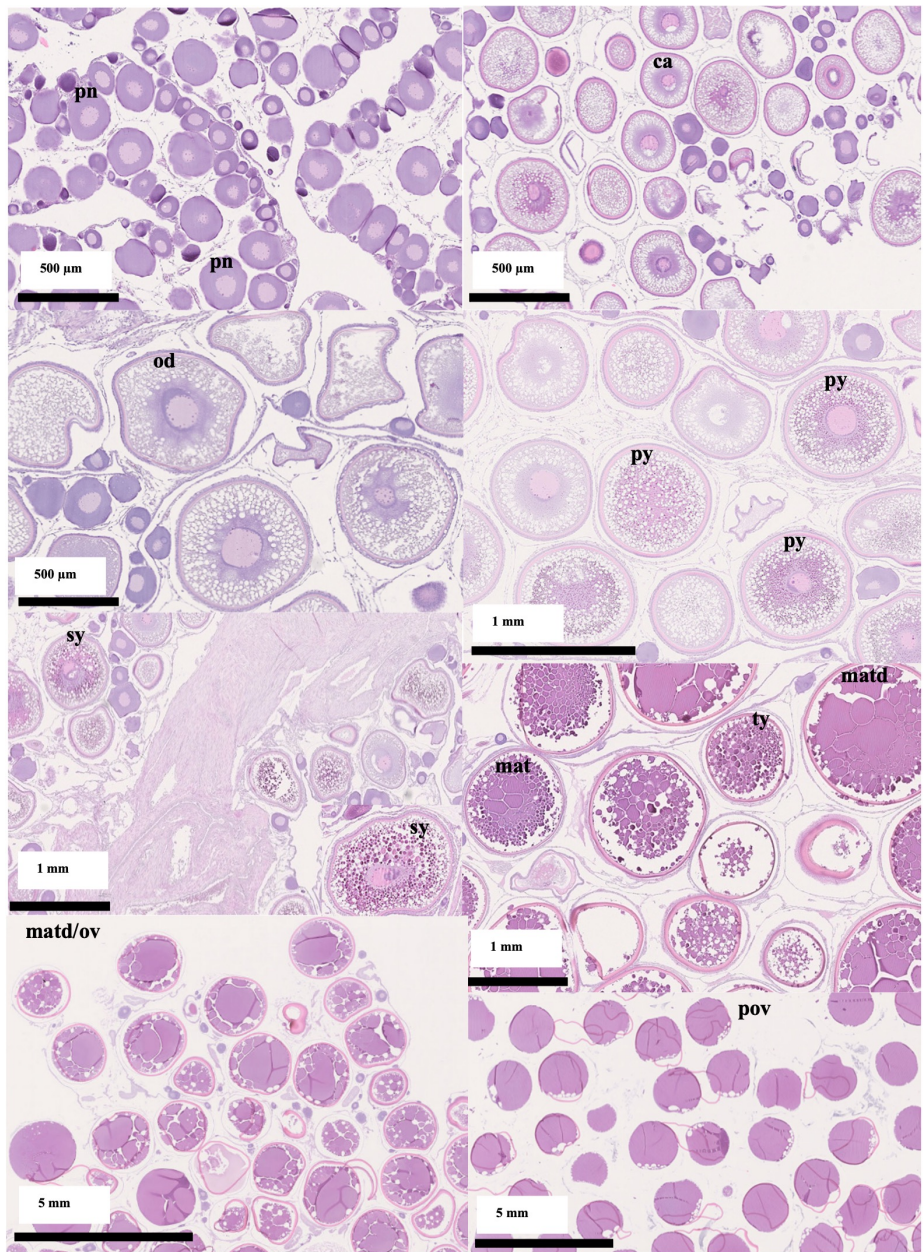

Supplement: S7 Fig — 10 stages were identified: pn, perinucleolus; ca, cortical alveoli; od, oil droplet; py, primary yolk; sy, secondary yolk; ty, tertiary yolk; mat, maturing; matd, matured oocytes and ov, ovulating oocytes; pov, post-ovulatory eggs. Scale bars are shown in the Figure. (PDF) [file pone.0311735.s007.pdf]

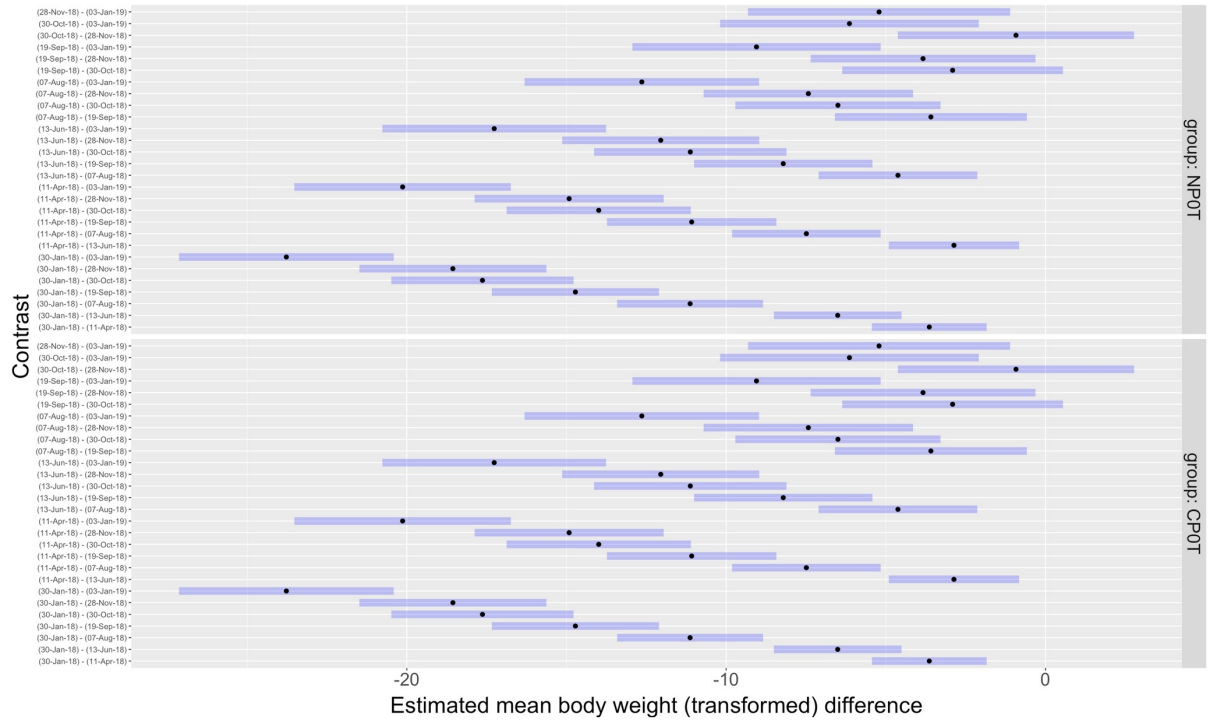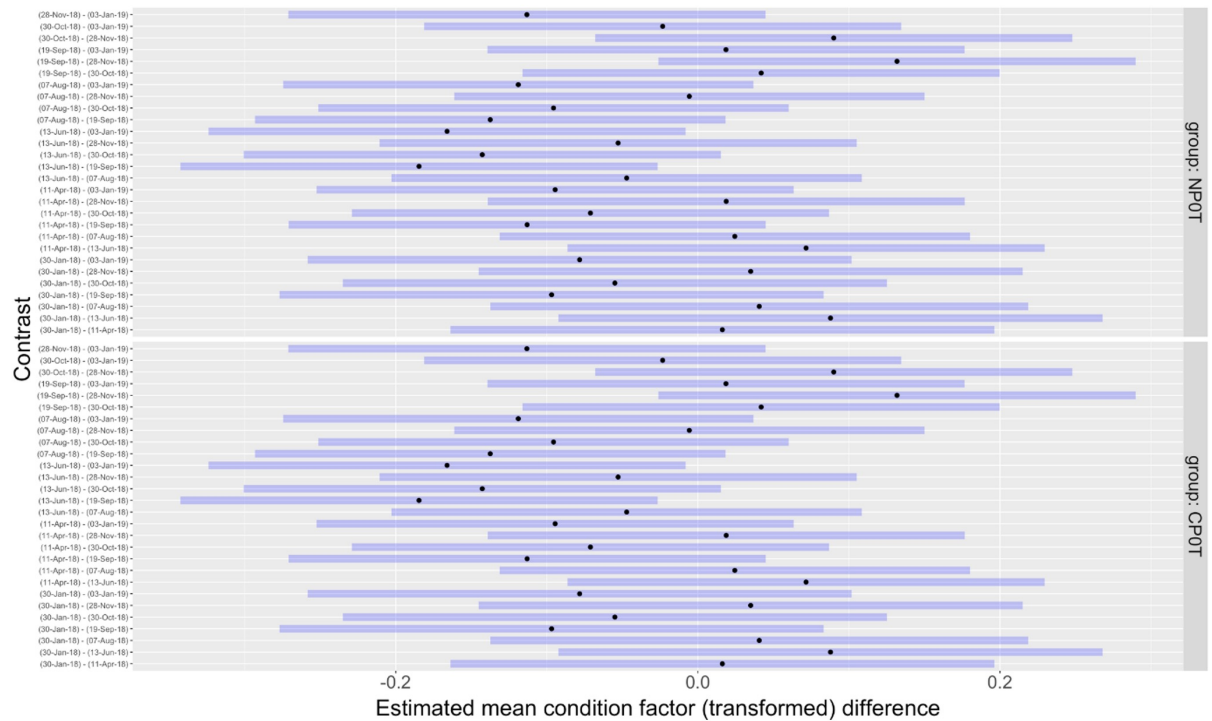

Supplement: S8 Fig — There are separate panels for each group, representing estimated mean differences between sampling points. Within each panel, the contrasts between sampling points suggesting temporal variations in the body weight and condition factor are shown. Contrasts where blue bars do not cross the zero exhibit statistically significant differences, indicating varying impacts of time on body weight and condition factor in the different groups. (PDF) [file pone.0311735.s008.pdf]

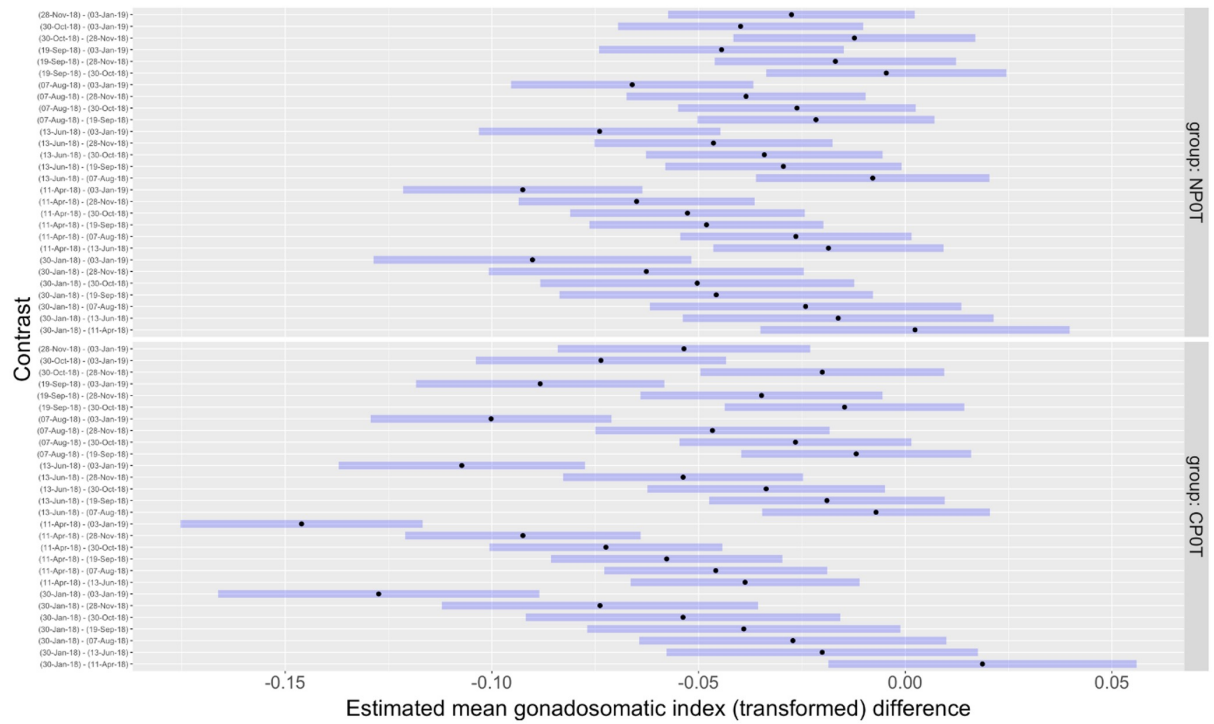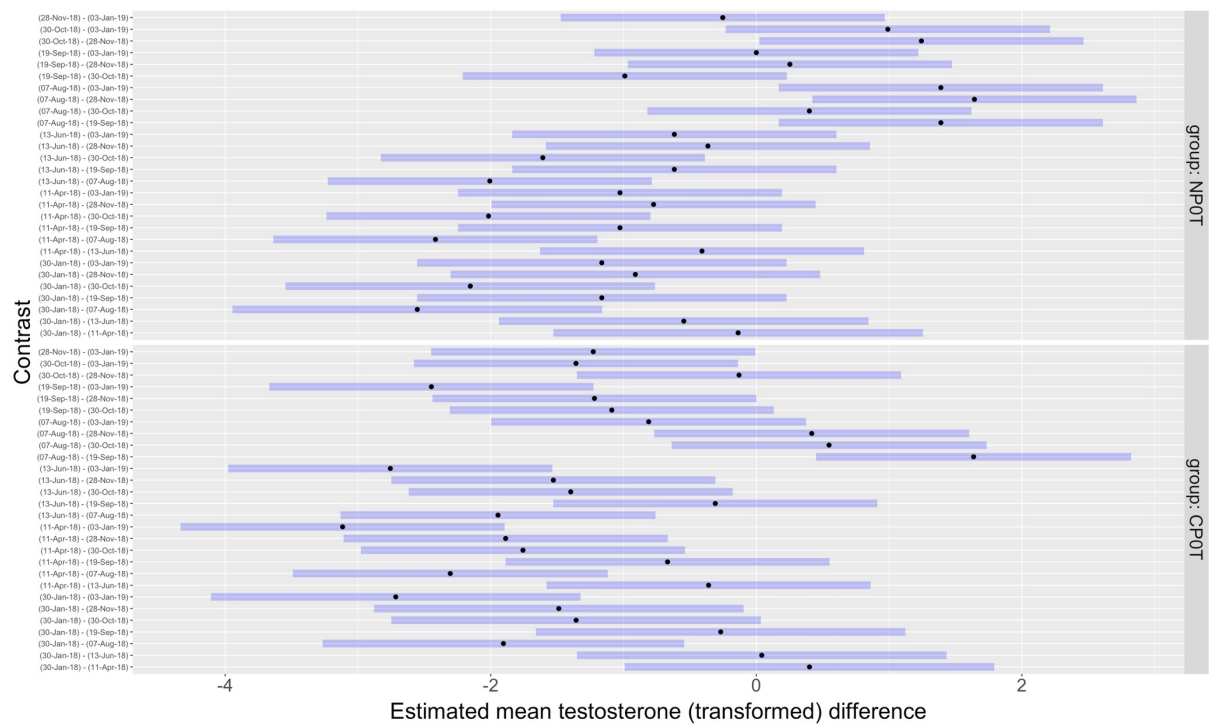

Supplement: S9 Fig — There are separate panels for each group, representing estimated mean differences between sampling points. Within each panel, the contrasts between sampling points suggesting temporal variations in gonadosomatic index and testosterone are shown. Contrasts where blue bars do not cross the zero exhibit statistically significant differences, indicating varying impacts of time on gonadosomatic index and testosterone in the different groups. (PDF) [file pone.0311735.s009.pdf]

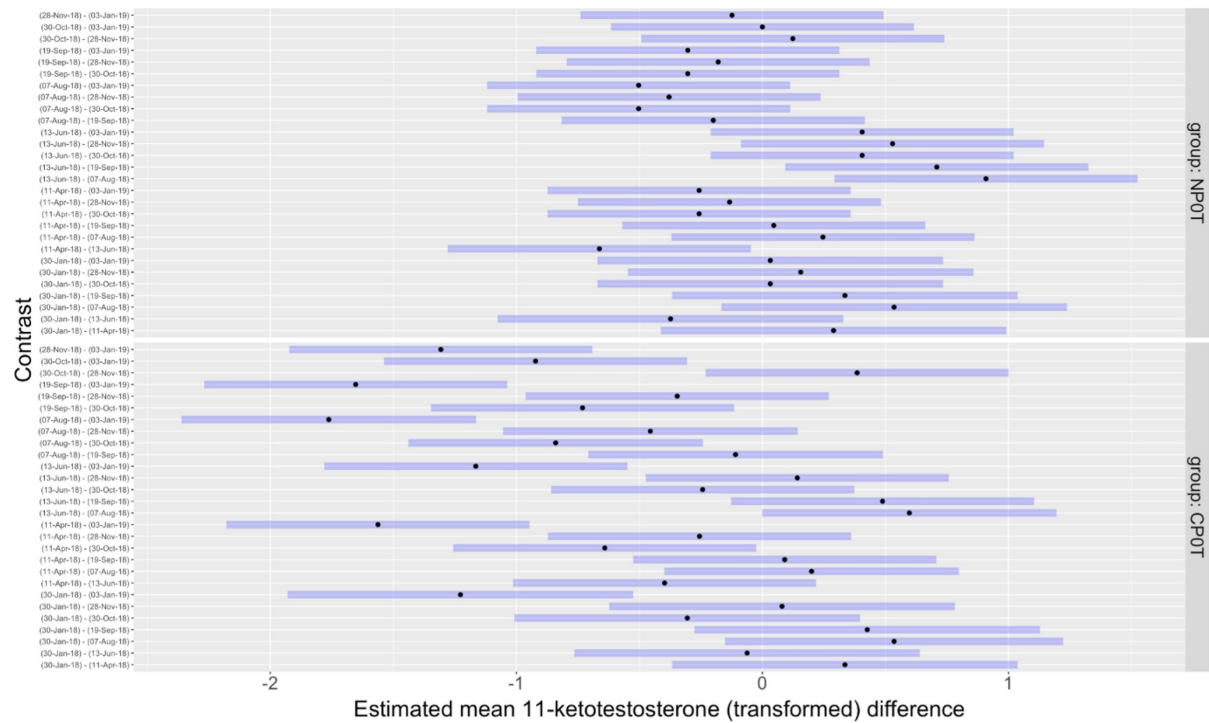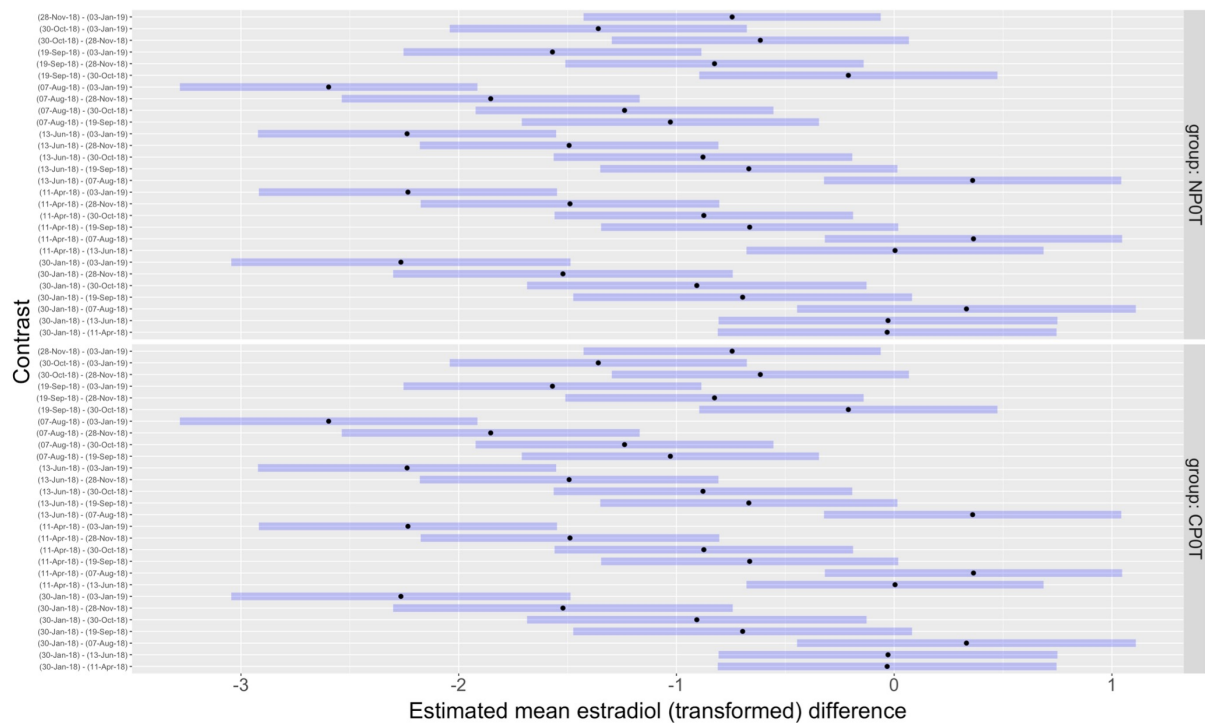

Supplement: S10 Fig — There are separate panels for each group, representing estimated mean differences between sampling points. Within each panel, the contrasts between sampling points suggesting temporal variations in the 11-ketotestosterone and 17β-estradiol are shown. Contrasts where blue bars do not cross the zero exhibit statistically significant differences, indicating varying impacts of time on 11-ketotestosterone and 17β-estradiol in the different groups. (PDF) [file pone.0311735.s010.pdf]

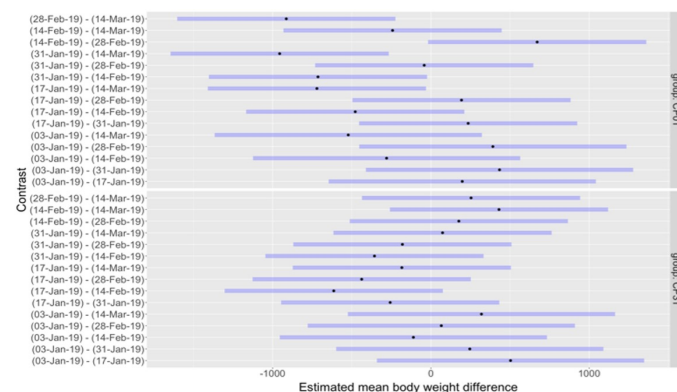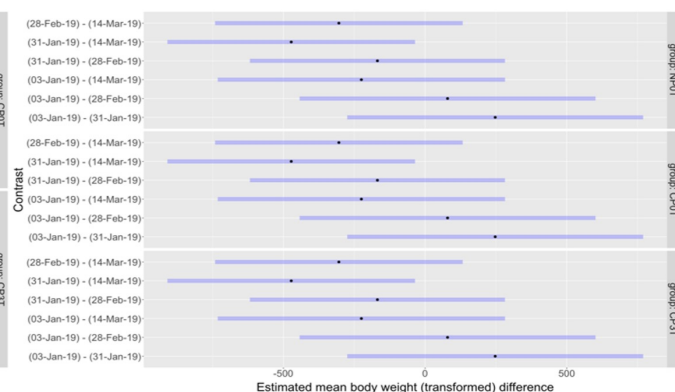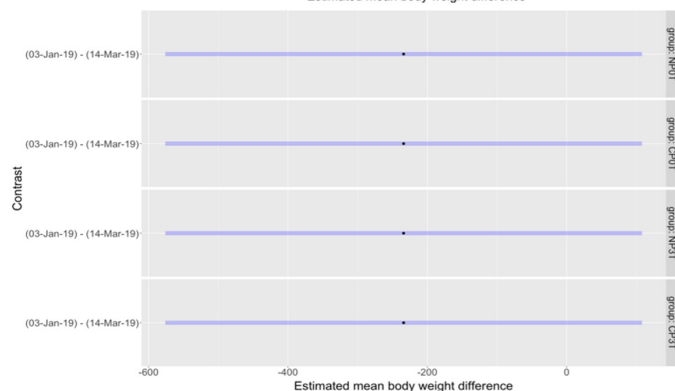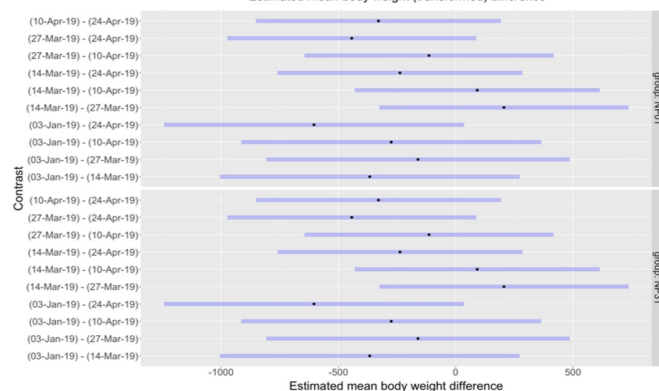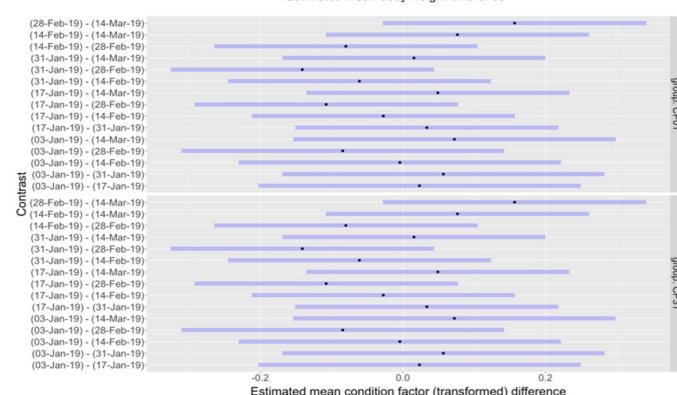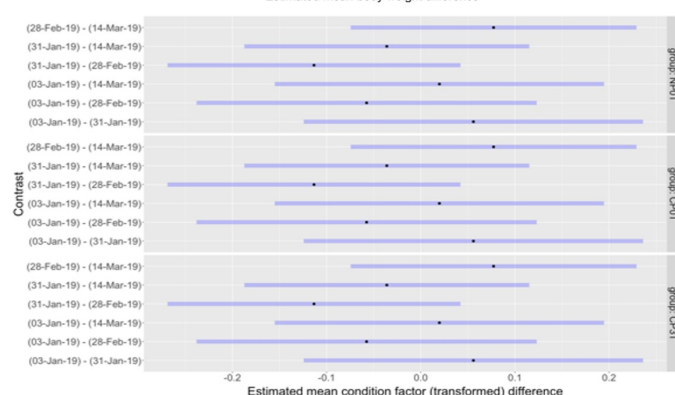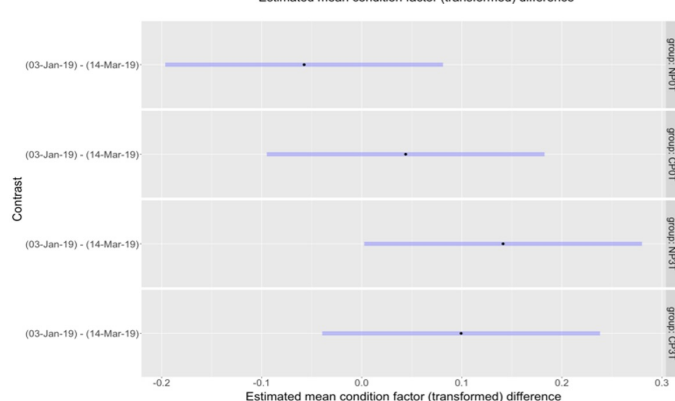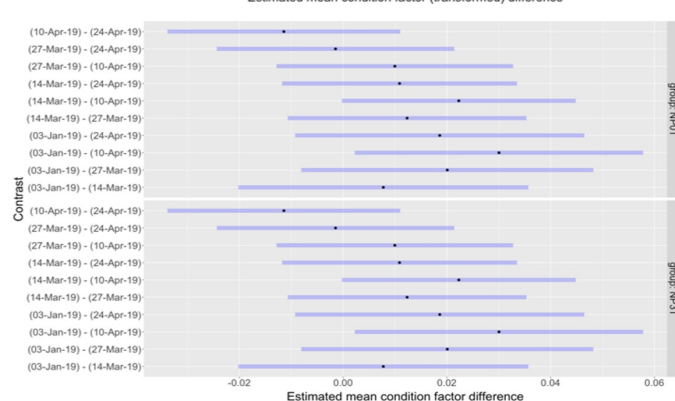

Supplement: S11 Fig — There are separate panels for each group, representing estimated mean differences between sampling points. Within each panel, the contrasts between sampling points suggesting temporal variations in the body weight and condition factor are shown. Contrasts where blue bars do not cross the zero exhibit statistically significant differences, indicating varying impacts of time on body weight and condition. (PDF) [file pone.0311735.s011.pdf]

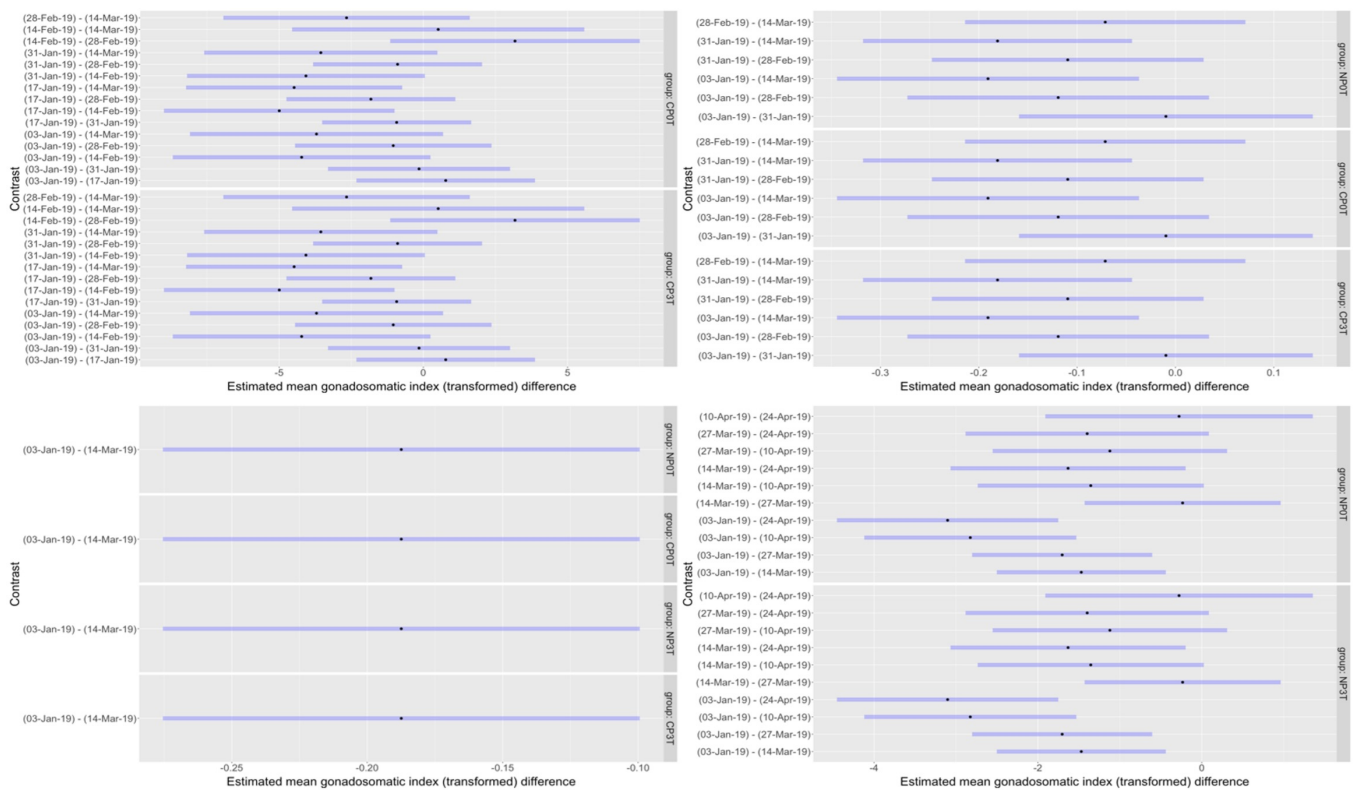

Supplement: S12 Fig — There are separate panels for each group, representing estimated mean differences between sampling points after temperature elevation. Within each panel, the contrasts between sampling points suggesting temporal variations in the gonadosomatic index are shown. Contrasts where blue bars do not cross the zero exhibit statistically significant differences, indicating varying impacts of time on gonadosomatic index in the different groups. (PDF) [file pone.0311735.s012.pdf]

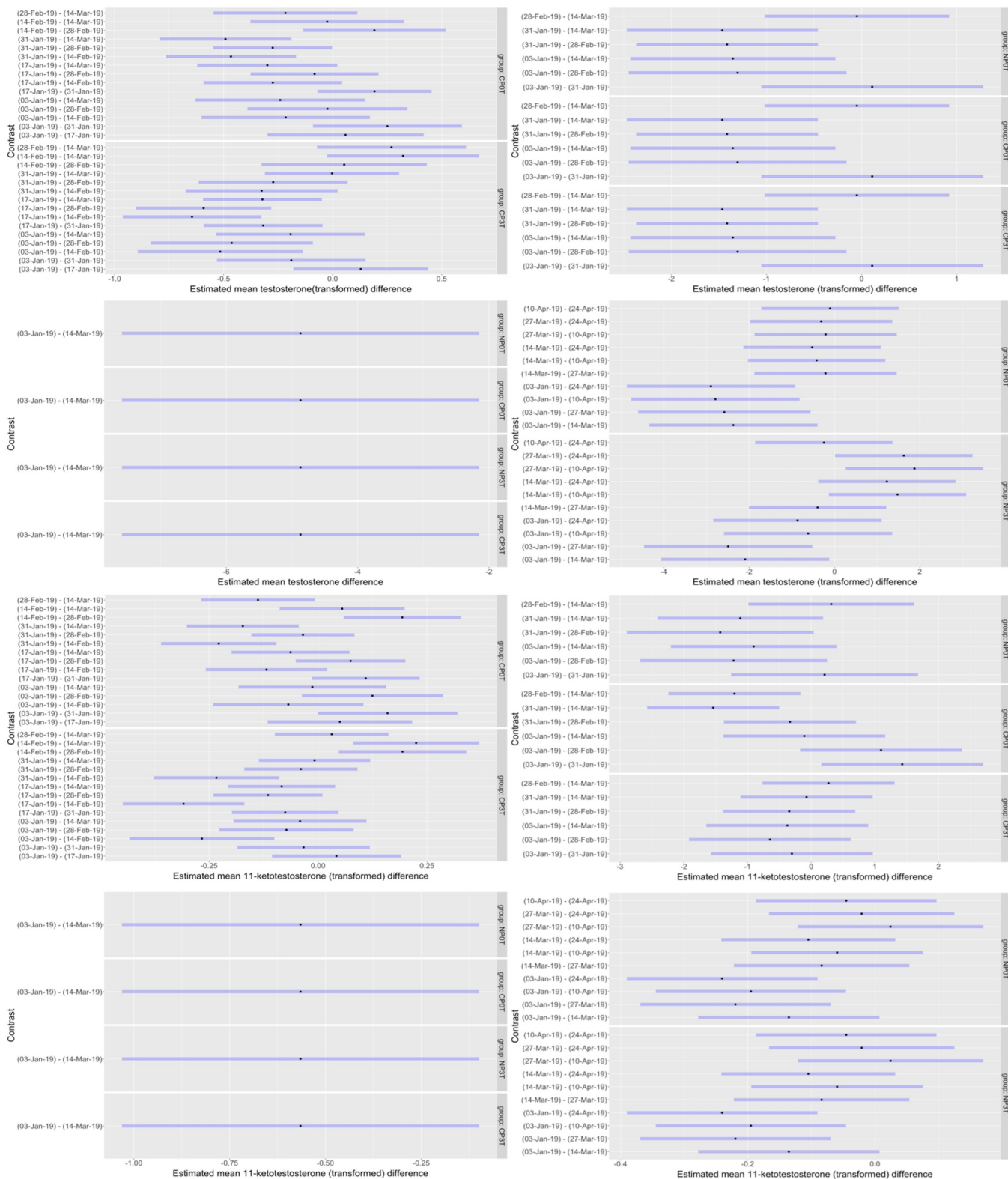

Supplement: S13 Fig — There are separate panels for each group, representing estimated mean differences between sampling points. Within each panel, the contrasts between sampling points suggesting temporal variations in the testosterone and 11-ketotestosterone are shown. Contrasts where blue bars do not cross the zero exhibit statistically significant differences, indicating varying impacts of time on testosterone and 11-ketotestosterone in the different groups. (PDF) [file pone.0311735.s013.pdf]

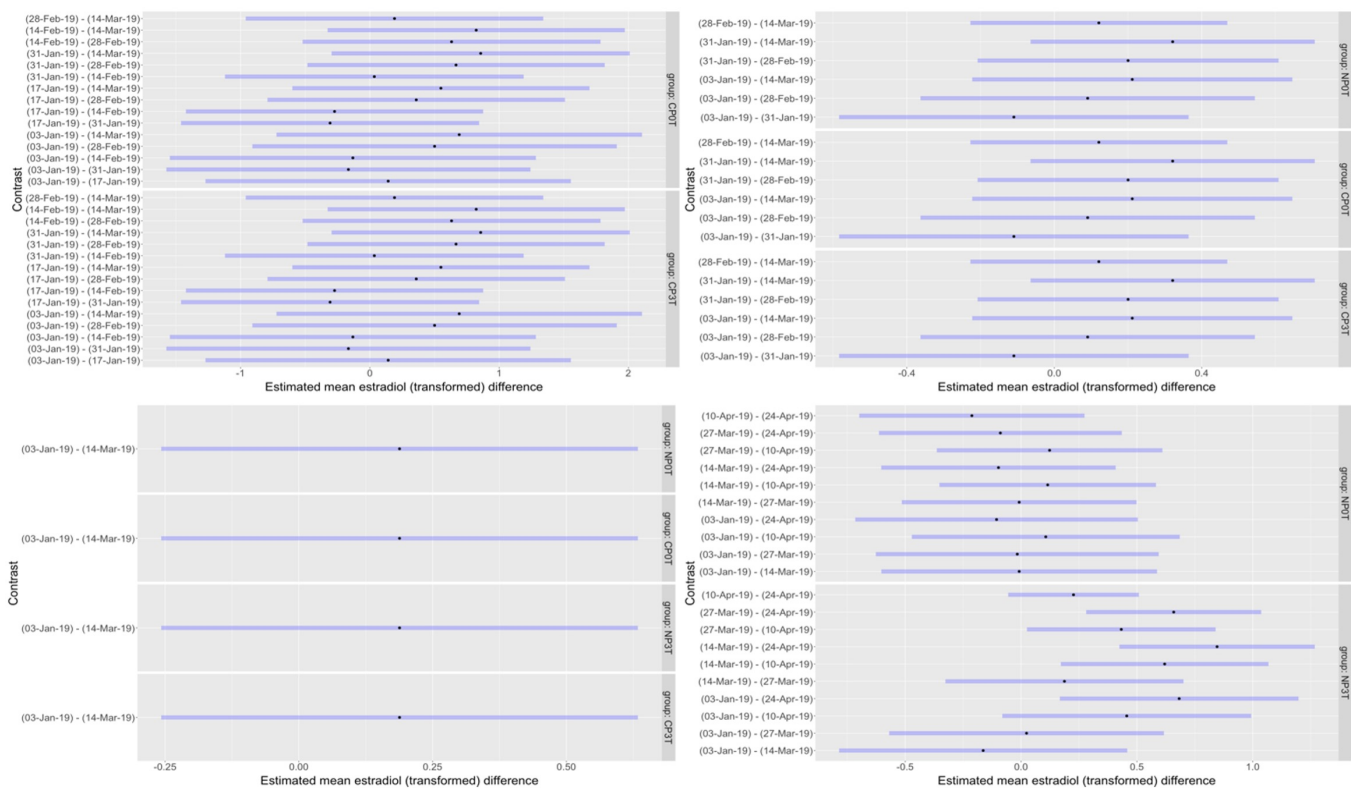

Supplement: S14 Fig — There are separate panels for each group, representing estimated mean differences between sampling points. Within each panel, the contrasts between sampling points suggesting temporal variations in 17β-estradiol are shown. Contrasts where blue bars do not cross the zero exhibit statistically significant differences, indicating varying impacts of time on 17β-estradiol in the different groups. (PDF) [file pone.0311735.s014.pdf]
