# Supplementary material for: Reproductive performance of lumpfish (Cyclopterus lumpus, L. 1758) females: Effects of integrated photoperiod and temperature manipulations on sexual maturation and spawning
Source: PLoS One. 2024 Oct 15;19(10):e0311735. doi: 10.1371/journal.pone.0311735 (PMC11478831; doi:10.1371/journal.pone.0311735)
Supplement: S1 Table — The blue shaded region is the sampling period before temperature elevation, the orange shaded region is the sampling period after temperature elevation. In seven sampling points after temperature elevation, one to two groups were not sampled. (PDF) [file pone.0311735.s015.pdf]

Sampling Date	Group	Sample Size
30-Jan-2018	Initial	5
11-Apr-2018	NP0T	8
11-Apr-2018	CP0T	8
13-Jun-2018	NP0T	8
13-Jun-2018	CP0T	8
7-Aug-2018	NP0T	8
7-Aug-2018	CP0T	9
19-Sep-2018	NP0T	8
19-Sep-2018	CP0T	8
30-Oct-2018	NP0T	8
30-Oct-2018	CP0T	8
28-Nov-2018	NP0T	8
28-Nov-2018	CP0T	8
3-Jan-2019	NP0T	8
3-Jan-2019	CP0T	8
17-Jan-2019	CP0T	8
17-Jan-2019	CP3T	8
17-Jan-2019	NP0T*	0
17-Jan-2019	NP3T*	0
31-Jan-2019	NP0T	8
31-Jan-2019	CP0T	8
31-Jan-2019	CP3T	8
31-Jan-2019	NP3T*	0
14-Feb-2019	CP0T	8
14-Feb-2019	CP3T	8
14-Feb-2019	NP0T*	0
14-Feb-2019	NP3T*	0
28-Feb-2019	NP0T	8
28-Feb-2019	CP0T	8
28-Feb-2019	CP3T	8
28-Feb-2019	NP3T*	0
14-Mar-2019	NP0T	8
14-Mar-2019	CP0T	8
14-Mar-2019	NP3T	8
14-Mar-2019	CP3T	8
27-Mar-2019	NP0T	8
27-Mar-2019	NP3T	8
27-Mar-2019	CP0T*	0
27-Mar-2019	CP3T*	0
10-Apr-2019	NP0T	8
10-Apr-2019	NP3T	8
10-Apr-2019	CP0T*	0
10-Apr-2019	CP3T*	0
24-Apr-2019	NP0T	8
24-Apr-2019	NP3T	8
24-Apr-2019	CP0T*	0
24-Apr-2019	CP3T*	0
