# Supplementary material for: Reproductive performance of lumpfish (Cyclopterus lumpus, L. 1758) females: Effects of integrated photoperiod and temperature manipulations on sexual maturation and spawning
Source: PLoS One. 2024 Oct 15;19(10):e0311735. doi: 10.1371/journal.pone.0311735 (PMC11478831; doi:10.1371/journal.pone.0311735)
Supplement: S2 Table — The values for NP3T and CP3T are specific to the period after temperature elevation. Values are mean ± SD. (PDF) [file pone.0311735.s016.pdf]

Group	Temperature (℃)	Oxygen (%)
NP0T	4.16 ± 0.47	92.5 ± 4.35
CP0T	4.13 ± 0.5	93.12 ± 4.28
NP3T	6.96 ± 0.19	91.14 ± 3.01
CP3T	7 ± 0.17	89.56 ± 6.03
