# Supplementary material for: Reproductive performance of lumpfish (Cyclopterus lumpus, L. 1758) females: Effects of integrated photoperiod and temperature manipulations on sexual maturation and spawning
Source: PLoS One. 2024 Oct 15;19(10):e0311735. doi: 10.1371/journal.pone.0311735 (PMC11478831; doi:10.1371/journal.pone.0311735)
Supplement: Attachment — Submitted filename: To PLOS ONE Officials.docx [file pone.0311735.s018.docx]

Dear PLOS ONE Officials,

I am grateful for your consideration of the newly submitted manuscript, and I am happy to respond to the raised issues.

1. The currently submitted manuscript (PONE-D-24-25185) is not a resubmission of the previous manuscript (PONE-D-23-26339) for the following reasons:

a. The two manuscripts originate from different experiments with distinct objectives.

-The current manuscript (PONE-D-24-25185) is based on an 18-month long experiment aimed at studying the reproductive performance of lumpfish subjected to

different photothermal regimes (natural photoperiod and compressed photoperiod, integrated with temperature manipulations) from the juvenile stage. In this

study, we assessed sexual maturation and spawning in females.

-The previous manuscript (PONE-D-23-26339) was based on a six-month experiment with the objective of describing the gonadal development and associated

endocrine factors in pooled samples of lumpfish broodstock (both females and males) from different photothermal regimes (short-to-long photoperiod and

continuous photoperiod integrated with temperature manipulations). This manuscript, in its original version, will not be resubmitted to this journal.

-Although both experiments were financed by the same funder, they had different objectives and designs.

-The author lists in the two manuscripts are also different due to the different objectives and hence different author engagements.

2. Because the current manuscript (PONE-D-24-25185) is not a resubmission but a new submission and is unrelated to a previous consideration by the journal, I do not have track changes to upload.

Thank you for your attention to this matter. Please let me know if there are any further questions or clarifications needed.

Additionally, I would like to disclose that I am co-author of the following works from the same project:

1.) A Preprint (Withdrawn): “Reproductive biology of farmed lumpfish Cyclopterus lumpus: Gonadal development and associated profiles of selected endocrine factors” https://doi.org/10.1101/2023.06.16.545335. (The work has been withdrawn, making it unsuitable as a reference).

2.) The academic final project report to the Norwegian Seafood Research Fund (FHF), which provides an overview of all studies and activities in the CycloBreed project: “Reproductive biology of lumpfish (Cyclopterus lumpus): a key to successive selective breeding”. file:///Users/frankmlingi/Downloads/Sluttrapport_%20Reproductive%20biology%20of%20lumpfish%20(Cyclopterus%20lumpus)_%20A%20key%20to%20successful%20selective%20breeding%20(2).PDF.

3.) The scientific journal publication: “Ultrasonic imaging as a means of monitoring gonadal development in lumpfish (Cyclopterus lumpus)” https://doi.org/10.14814/phy2.15811. The publication is also available at the NTNU Open Archive (https://ntnuopen.ntnu.no/ntnu-xmlui/handle/11250/3093158) and the research institution “Nofima”: https://hdl.handle.net/11250/3092572, as well as other platforms to which the authors are affiliated.

4.) Conference abstracts:

A. “Photothermal control of sexual maturation in lumpfish (Cyclopterus lumpus)”. Aquaculture Europe 2021. https://aquaeas.org/MeetingAbstracts/PaperDetail/38951.

B. Gonad development and plasma levels of sex steroids in farmed lumpfish (Cyclopterus lumpus) under different photoperiod and temperature regimes. Aquaculture Europe 2019.
