# Supplementary material for: Reproductive performance of lumpfish (Cyclopterus lumpus, L. 1758) females: Effects of integrated photoperiod and temperature manipulations on sexual maturation and spawning
Source: PLoS One. 2024 Oct 15;19(10):e0311735. doi: 10.1371/journal.pone.0311735 (PMC11478831; doi:10.1371/journal.pone.0311735)
Supplement: Attachment — Submitted filename: Reviewers feedback on PONE-D-24-25185.docx [file pone.0311735.s019.docx]

**Reviewer Feedback**

**Title: Reproductive performance of lumpfish (Cyclopterus lumpus, L. 1758) females: Effects**

**of integrated photoperiod and temperature manipulations on sexual maturation and**

**spawning**

**Manuscript number: PONE-D-24-25185**

**Overall Recommendations: Minor Revision!**

***Section wise feedback***

1. **Title and Abstract**:
   - The title is clear and accurately reflects the content of the manuscript.
   - The abstract provides a concise summary of the study's aims, methods, results, and conclusions. It effectively highlights the significance of photoperiod and temperature manipulations on lumpfish reproduction.
2. **Introduction**:
   - The introduction provides a comprehensive background on the importance of lumpfish in aquaculture and the need for controlled reproduction. However, it could benefit from a more detailed explanation of the current challenges in lumpfish breeding and how this study addresses those gaps.
3. **Materials and Methods**:
   - The experimental design is robust, and the methods are described in detail, allowing for reproducibility. The methodology section is comprehensive, but it would be helpful to include a flowchart summarizing the experimental design, treatments, and key measurements. This visual aid can enhance clarity for readers.
4. **Results**:
   - The results are detailed and well-organized, but the inclusion of more graphical representations (e.g., line charts or bar graphs) could make complex data trends more accessible.
   - When discussing significant findings, consider adding more context about the biological significance of these results. For example, explain why certain hormone levels fluctuated and how this impacts lumpfish reproduction.
   - Consider providing more detailed explanations for the observed fluctuations in body weight and condition factor after temperature elevation.
5. **Discussion**:
   - The discussion effectively interprets the results in the context of existing literature.
   - The discussion is thorough, but it would benefit from a clearer connection between the study’s findings and their practical implications for lumpfish aquaculture. How can these results be applied to improve breeding practices?
   - Address any limitations of the study more explicitly and suggest areas for future research to provide a balanced perspective.
6. **Conclusion**:

Highlighting how these findings can be implemented in aquaculture settings would emphasize the study's relevance.
