# Supplementary material for: Reproductive performance of lumpfish (Cyclopterus lumpus, L. 1758) females: Effects of integrated photoperiod and temperature manipulations on sexual maturation and spawning
Source: PLoS One. 2024 Oct 15;19(10):e0311735. doi: 10.1371/journal.pone.0311735 (PMC11478831; doi:10.1371/journal.pone.0311735)
Supplement: Attachment — Submitted filename: Response to Reviewer Feedback on PONE-D-24-25185.docx [file pone.0311735.s020.docx]

**Reviewer Feedback**

**Title: Reproductive performance of lumpfish (Cyclopterus lumpus, L. 1758) females: Effects**

**of integrated photoperiod and temperature manipulations on sexual maturation and**

**spawning**

**Manuscript number: PONE-D-24-25185**

**Overall Recommendations: Minor Revision!**

***Section wise feedback***

1. **Title and Abstract**:
   - The title is clear and accurately reflects the content of the manuscript.
   - The abstract provides a concise summary of the study's aims, methods, results, and conclusions. It effectively highlights the significance of photoperiod and temperature manipulations on lumpfish reproduction.
2. **Introduction**:
   - The introduction provides a comprehensive background on the importance of lumpfish in aquaculture and the need for controlled reproduction. However, it could benefit from a more detailed explanation of the current challenges in lumpfish breeding and how this study addresses those gaps.

**Thank you very much for your suggestion. In response, we have added a paragraph explaining several traits and the associated challenges in lumpfish as cleaner fish. Additionally, we have explained how breeding programs aim to address these challenges and how our study can contribute to successful development of such programs. The paragraph has been positioned to ensure a smooth transition to the subsequent sections, which focus on the specific aims of this study.**

3. **Materials and Methods**:
   - The experimental design is robust, and the methods are described in detail, allowing for reproducibility. The methodology section is comprehensive, but it would be helpful to include a flowchart summarizing the experimental design, treatments, and key measurements. This visual aid can enhance clarity for readers.

**Thank you very much for your suggestion. In response, we have included a flowchart that outlines the experiment, sampling, and data collection process as a figure.**

4. **Results**:
   - The results are detailed and well-organized, but the inclusion of more graphical representations (e.g., line charts or bar graphs) could make complex data trends more accessible.

**Thank you very much for your suggestion. We acknowledge that, due to the nature of the experimental design and some unexpected events, the data trends are indeed complex. To aid in understanding these trends, we included line charts in the initial submission (Figures 5 and 6). In this revision, we have added supplementary line graphs for body weight, condition factor, gonadosomatic index and sex steroids presenting only the mean values. These additional graphs are included in the supplementary materials to avoid what we felt could be duplicate data visualizations. We hope this approach will enhance understanding for interested readers.**

- When discussing significant findings, consider adding more context about the biological significance of these results. For example, explain why certain hormone levels fluctuated and how this impacts lumpfish reproduction.
- **Thank you for your feedback. We have added possible explanations regarding the biological significance of variations in the sexual maturation indicators to enhance relevant discussions in the discussion section, particularly in relation to photoperiod and temperature.**
- Consider providing more detailed explanations for the observed fluctuations in body weight and condition factor after temperature elevation.

**Thank you for your suggestion. We have added more explanations regarding the fluctuations in body weight and condition factor in the respective sections.**

5. **Discussion**:
   - The discussion effectively interprets the results in the context of existing literature.
   - The discussion is thorough, but it would benefit from a clearer connection between the study’s findings and their practical implications for lumpfish aquaculture. How can these results be applied to improve breeding practices?

**Thank you for your suggestion. A paragraph highlighting the practical implications of the study’s findings on lumpfish aquaculture has been added. This paragraph discusses how the findings related to photothermal manipulations can be implemented and optimized in lumpfish juvenile production. Furthermore, we have addressed the need for additional research aimed at optimizing these photothermal manipulations and enhancing the efficiency of breeding programs.**

- Address any limitations of the study more explicitly and suggest areas for future research to provide a balanced perspective.

**Thank you for your suggestion. We have highlighted limitations of the study related to its nature, small sample sizes, fish sacrifice and atypical furunculosis challenge in the discussion. Additionally, we have suggested considerations for future studies regarding sample size and sampling frequency, as well as replacing fish sacrifice with less invasive methods. This paragraph has been positioned towards the end of the discussion.**

6. **Conclusion**:

- Highlighting how these findings can be implemented in aquaculture settings would emphasize the study's relevance.

**Thank you for your feedback. We have highlighted how the findings can be implemented in aquaculture settings. Additionally, we highlight how breeding programs and commercial lumpfish farms may benefit from the photothermal manipulations.**
